# Maize ZmbZIP92 transcription factor positively regulates drought tolerance in *Arabidopsis*

**DOI:** 10.1080/15592324.2026.2635681

**Published:** 2026-02-26

**Authors:** Zukuan Liu, Jian Shang, Min Liu, Jietian Kong, Aiya Huang, Yang Zhao, Qing Ma, Wei Dai

**Affiliations:** a National Engineering Laboratory of Crop Stress Resistance Breeding, School of Life Sciences, Anhui Agricultural University, Hefei, People's Republic of China; b Engineering Research Center for Maize of Anhui Province, School of Life Sciences, Anhui Agricultural University, Hefei, People's Republic of China

**Keywords:** Drought tolerance, Maize, *ZmbZIP92*, transcription factor, ABA

## Abstract

Plants are susceptible to various environmental stresses, but basic leucine zipper (bZIP) transcription factors play a key role in regulating stress responses. In this study, a drought response-related candidate gene (*ZmbZIP92*) was cloned from maize (*Zea mays* L.) following a comparative genomic analysis. This gene is highly homologous to the rice (*Oryza sativa* L.) gene *OsbZIP62*, exhibits tissue-specific expression patterns, and is significantly induced by drought, high salinity, and abscisic acid (ABA) treatments. Subcellular localization revealed that ZmbZIP92 is a nuclear protein. Additionally, yeast-based assays of ZmbZIP92 detected a lack of transcriptional self-activation. Dual-luciferase reporter assays demonstrated that ZmbZIP92 binds specifically to the G-box (CACGTG) cis-element. Overexpressing *ZmbZIP92* in *Arabidopsis thaliana* significantly promoted root elongation, enhanced drought tolerance, and increased sensitivity to ABA, which was reflected by markedly inhibited seed germination. RNA sequencing and differential expression analyses indicated that multiple stress response-related pathways were enriched in *ZmbZIP92*-overexpressing plants, including ABA signaling, antioxidant response, transmembrane transport, and general plant signal transduction pathways. In summary, ZmbZIP92 positively regulates drought tolerance through its effects on the ABA signaling pathway and other stress response-related signaling networks.

## Article highlights

- ZmbZIP92 was identified as a novel maize bZIP transcription factor encoded by a drought- and ABA-inducible gene.
- ZmbZIP92 is localized to the nucleus and binds specifically to the G-box (CACGTG) element, but lacks transcriptional self-activation activity in yeast.
- *ZmbZIP92* overexpression in *Arabidopsis* enhances drought tolerance and confers increased sensitivity to ABA.
- ZmbZIP92 positively regulates drought responses, likely by modulating ABA signaling and stress-responsive gene expression.

## Introduction

Water, which constitutes approximately 80%–90% of the plant body, is essential for all physiological processes.^1^ Drought decreases the soil moisture content in agricultural systems, with the resulting impaired crop growth and development decreasing yield and quality.^2^^,^^3^ To adapt to the uneven spatiotemporal distribution and varying intensity of natural precipitation, plants have evolved diverse regulatory mechanisms involving morphological, physiological, and molecular processes.^4^ In recent years, substantial progress has been made in elucidating the molecular basis of plant drought responses and in identifying genes that regulate stress resistance.^5^^,^^6^ Consequently, improving plant drought tolerance has become a key focus in abiotic stress research and breeding for crop stress resilience.^7^

Transcription factors regulate target gene expression by binding to specific DNA motifs in promoter regions. Numerous transcription factor families, including MYB, WRKY, NAC, AP2/ERF, and basic leucine zipper (bZIP), mediate plant responses to environmental stress.^8^ In eukaryotes, bZIP family members are one of the most widely distributed and evolutionarily conserved transcription factors.^9^ Notably, they are activated by various abiotic stresses, including salinity, drought, cold, and heavy metals.^9^^,^^10^ In rice (*Oryza sativa* L.), OsbZIP62 and OsbZIP66 enhance drought tolerance through the abscisic acid (ABA) signaling pathway,^11–13^ whereas OsbZIP71 binds to the promoters of abiotic stress response-related genes and improves salt tolerance.^14^ In *Arabidopsis thaliana* (Arabidopsis), silencing *HY5*, which encodes a key component of the light signaling pathway, leads to increased sensitivity to salinity.^15^ Additionally, AtbZIP63 mediates glucose–ABA crosstalk under stress conditions.^16^ In the complex network of epigenetic and hormonal interactions, transcription factors serve as critical hubs that directly decode signals, such as ABA, to activate downstream stress-responsive genes, highlighting the importance of elucidating their functions.^17^

ABA is the central phytohormone regulating plant adaptations to abiotic stress.^18^ The canonical ABA-dependent signaling pathway has been thoroughly characterized. Specifically, stress-induced ABA accumulation promotes the binding of PYR/PYL/RCAR receptors to clade A PP2C phosphatases, thereby relieving their repressive effects on SnRK2 kinases.^19^^,^^20^ The resulting activated SnRK2s phosphorylate downstream transcription factors, including extensively studied bZIP family members. Comparative genomic analysis of maize identified 125 *bZIP* genes that encode 170 distinct proteins; they were phylogenetically classified into 11 subfamilies on the basis of their homology to *Arabidopsis* and rice sequences.^21^ A previous study showed that overexpressing subfamily D member *ZmbZIP4* increases ABA levels to enhance stress tolerance.^22^ Additionally, ZmbZIP76 in the F subclass of the bZIP family can bind to ACGT elements to enhance reactive oxygen species (ROS) scavenging and improve plant tolerance to salt and osmotic stresses.^23^ Subfamily S member ZmbZIP72 modulates abiotic stress responses through the ABA-dependent pathway,^24^ whereas subfamily I member ZmbZIP113 influences seed germination at low temperatures.^25^ Although bZIP transcription factors have been broadly characterized, the functions of subfamily I members in maize remain largely unexplored, leaving their potential role in abiotic stress responses an open question.

In this study, ZmbZIP92 was characterized as a drought- and ABA-inducible bZIP transcription factor that binds specifically to G-box elements. To dissect the potential molecular function of ZmbZIP92 within a controlled genetic background, the model plant *Arabidopsis* was used for heterologous expression analysis. The advantages of *Arabidopsis* include the availability of a fully sequenced genome, a mature genetic manipulation system, and a high degree of standardization in physiological assays, making it ideal for preliminary gene functional analysis and in-depth mechanistic investigations in plants.^26^^,^^27^ This strategy has been widely employed to elucidate the functions of stress-responsive genes in crops and reveal conserved regulatory mechanisms.^28^ In the current study, *ZmbZIP92* overexpression in *Arabidopsis* enhanced drought tolerance by improving ROS scavenging. This study provides valuable insights into a key molecular mechanism underlying drought stress adaptation as well as a promising genetic resource for breeding drought-resilient maize varieties.

## Materials and methods

### Plant materials and growth conditions

The plant materials used in this study were as follows: B73 homozygous maize inbred line, wild-type (WT) *Arabidopsis* Columbia (Col-0) ecotype, and *Nicotiana benthamiana*. All plant materials were provided by the National Engineering Laboratory for Crop Stress Resistance Breeding. Maize plants were cultivated in a greenhouse set at 28 ± 2 °C with a 14-h light/10-h dark photoperiod. *Arabidopsis* plants were grown under a 24 °C (16-h light)/22 °C (8 h dark) cycle. Tobacco plants were maintained at 24 °C with a 16-h light/8-h dark cycle. In the *Arabidopsis* root elongation assay, 200 and 300 mM mannitol were used to simulate drought conditions.

### Phylogenetic analysis of *ZmbZIP92*

On the basis of homology with the rice OsbZIP62 protein sequence in the NCBI database (https://www.ncbi.nlm.nih.gov/), *ZmbZIP92* was identified as a homologous gene in maize. Its genomic and coding sequences were retrieved from MaizeGDB (https://maizegdb.org/) and Phytozome V13 (https://phytozome-next.jgi.doe.gov/). Homologous protein sequences from related species were downloaded from NCBI. Multiple sequences were aligned using MEGA 6.0 software, after which a phylogenetic tree was constructed according to the maximum likelihood method. Topological robustness was assessed using 1,000 bootstrap replicates to quantify branch support. The multiple sequence alignment was visualized using the default parameters of DNAMAN 6.0 to highlight conserved functional domains.

### Plant transformation

The full-length *ZmbZIP92* coding region was cloned into the p1301a vector to construct the 35S::ZmbZIP92 overexpression vector. WT *Arabidopsis* (Col-0) was transformed using an *Agrobacterium*-mediated floral dip method. Harvested T_0_ seeds were screened for positive transgenic plants on half-strength solid Murashige and Skoog (MS) medium containing 20 mg/L hygromycin B. *ZmbZIP92* expression in transgenic lines was detected by quantitative real-time PCR (qRT-PCR) using primers qzmbzip92-F/R (Table S1) and *AtActin* as the internal reference gene.

### RNA extraction and qRT-PCR analysis

Total RNA was extracted from maize or *Arabidopsis* tissues using TRIzol reagent (Huamaike Bio, Beijing, China). Purified RNA and an Evo M-MLV RT Mix Kit (Accurate Biology, Changsha) were used to synthesize cDNA via reverse transcription. The subsequent qRT-PCR analysis was performed using a LightCycler 480 system. Relative gene expression levels were calculated according to the 2^−ΔΔCt^ method. Statistical significance was determined by one-way ANOVA and Tukey’s *post-hoc* test, which were completed using GraphPad Prism version 9.1.1 (Graph-Pad, San Diego, CA, USA). All experiments were conducted using three biological replicates.

### Subcellular localization of *ZmbZIP92*

The *ZmbZIP92* coding region without a stop codon was fused in-frame with the green fluorescent protein (GFP)-encoding sequence to construct the 35S::ZmbZIP92-GFP/pCAMBIA1305 vector. The recombinant vector was inserted into *Agrobacterium tumefaciens* strain GV3101 (p19) cells for the subsequent infiltration of *N. benthamiana* cells on the abaxial side of leaves. After a 36-h incubation in darkness, GFP fluorescence in the transiently transformed leaves was observed using an LSM800 laser scanning confocal microscope.

### Transcriptional self-activation assay of *ZmbZIP92*

The full-length *ZmbZIP92* coding region was cloned into the yeast expression vector pGBKT7. Y2HGold yeast competent cells were transformed with the recombinant vector (pGBKT7-ZmbZIP92). Transformants were sequentially plated on solid SD/−Trp and SD/−Trp/−His/−Ade (containing X-*α*-gal) media, which were incubated at 30 °C for 3–5 days to monitor growth and color development. pGBKT7-53 and pGADT7-T were used as positive controls, whereas pGBKT7-Lam and pGADT7-T were used as negative controls.

### Dual-luciferase reporter assay

The *ZmbZIP92* coding region was cloned into the pGreenII 62-SK vector to serve as the effector. A fragment containing four tandem repeats of the G-box element (CACGTG) was inserted into the pGreenII 0800-LUC vector as the reporter. Effector and reporter vectors were used for the co-transformation of *A. tumefaciens* strain GV3101 (pSoup-p19) cells for the subsequent co-infiltration of *N. benthamiana* leaves. With the empty pGreenII 0800-LUC vector serving as a control, luciferase activity was measured at 36 h post-infiltration using a dual-luciferase reporter assay kit and a Tanon-5200 *in vivo* imaging system.

### ABA sensitivity assay

Surface-sterilized seeds of transgenic and WT *Arabidopsis* were sown evenly on half-strength solid MS medium supplemented with 0, 0.5, or 1 μM ABA in plates. Each treatment was performed in triplicate. Plates were placed horizontally and cultured for 7 days, after which the seed germination rate was recorded. Data are presented herein as the mean ± standard deviation of at least three biological replicates (sample size ≥ 50).

### Physiological analysis

Peroxidase (POD) activities and malondialdehyde (MDA) contents were determined using commercial assay kits (Solarbio, Beijing, China). The relative water content (RWC) of leaves was measured using a gravimetric method and the following formula: RWC (%) = [(fresh weight − dry weight)/(turgid weight − dry weight)] × 100. To determine the turgid weight, leaves were immersed in water for 8–10 h and then carefully blotted to remove surface moisture before weighing. Three biological replicates were used in each group.

### RNA sequencing and analysis

Seeds of WT and *ZmbZIP92*-overexpressing line L5 *Arabidopsis* plants were surface-sterilized and sown on half-strength solid MS medium. After a 10-day incubation, seedlings were transplanted in nutrient soil. Following a 14-day cultivation, seedlings were exposed to drought stress. Leaves at the same developmental stage were collected (three biological replicates per group), flash-frozen in liquid nitrogen, and stored at −80 °C. Sequencing was performed using an Illumina NovaSeq 6000 system (LC Bio Technology Co., Ltd. Hangzhou, China). Differentially expressed genes (DEGs) were identified on the basis of FPKM values and the following thresholds: |log_2_(fold-change)| > 1 and *p* < 0.05.

### Statistical analysis

Physiological and biochemical data were processed using GraphPad Prism 9.0 software (Graph-Pad). Data are presented herein as the mean ± standard deviation of at least three biological replicates (n ≥ 3). Statistical significance (^∗^*p* < 0.05; ^∗∗^*p* < 0.01) was determined by Student’s *t*-test and one-way ANOVA.

## Results

### *ZmbZIP92* was identified as a novel *bZIP* gene in maize

**Figure 1.**
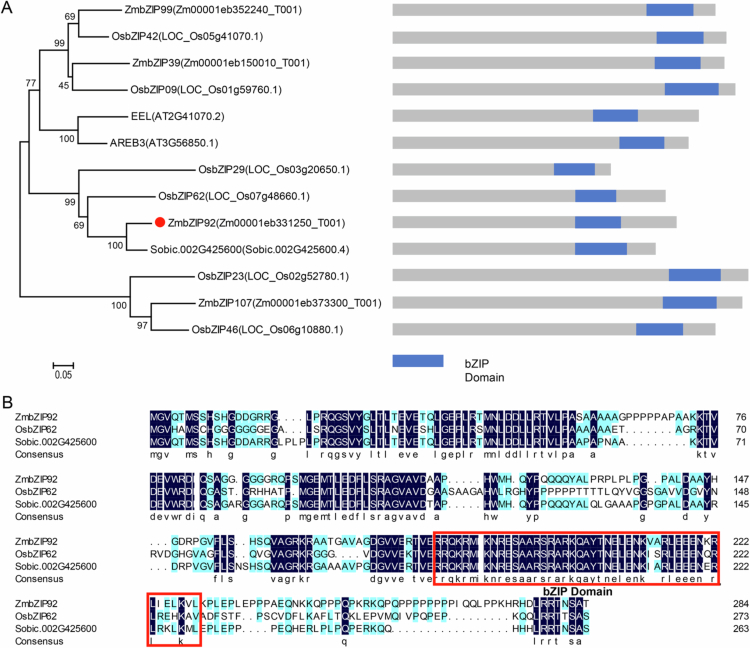
Phylogenetic tree of ZmbZIP92 and related bZIP proteins. (A) Phylogenetic tree of ZmbZIP92 and homologous bZIP proteins from various plant species. The tree was constructed by the neighbor-joining method with 1,000 bootstrap replicates using MEGA 7.0. All protein sequences were obtained from Phytozome v14.0. The blue regions indicates the conserved bZIP domain. (B) Multiple sequence alignment of amino acid sequences. The conserved bZIP and DNA-binding domains are indicated by solid lines beneath the sequences.

Considering the reported contribution of *OsbZIP62* to the rice response to drought stress,^12^ its homolog in maize was identified (i.e., *ZmbZIP92*) on the basis of comparative genomic analysis (Figure 1A). The encoded protein, which was classified in subgroup I among the 11 bZIP subclasses in maize, was revealed to contain a basic region for binding DNA and a leucine zipper domain that mediates protein dimerization. According to the constructed phylogenetic tree, maize *ZmbZIP92* is closely related to rice *OsbZIP62* and sorghum *Sobic-002G425600* (Figure 1B), suggesting that *ZmbZIP92* may encode a regulator of maize drought tolerance.

### *ZmbZIP92* expression pattern analysis

**Figure 2.**
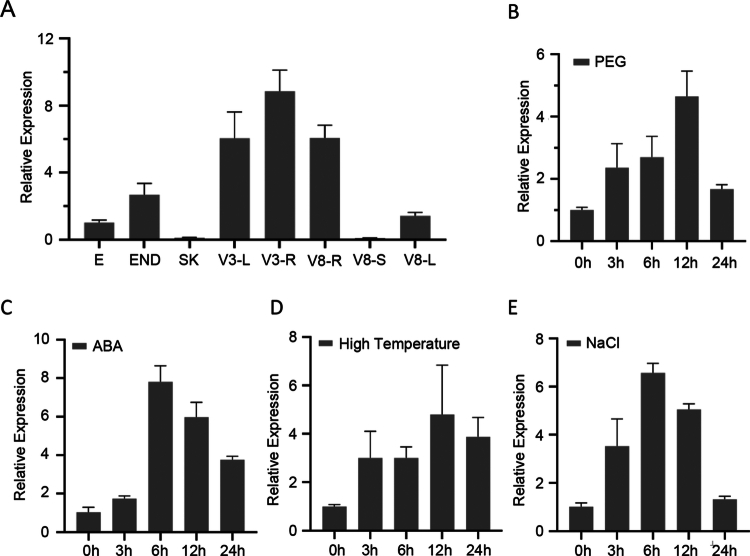
Expression patterns of *ZmbZIP92*. (A) Tissue-specific expression of *ZmbZIP92* at vegetative (V3: roots and leaves; V8: roots, stems, and leaves) and reproductive (SK: silks; E: embryo; END: endosperm) stages. (B-E) Expression of *ZmbZIP92* under various abiotic stress treatments: simulated drought (20% PEG-6000), 100 μM ABA, heat (42 °C), and high salinity (200 mM NaCl). Samples were collected at the indicated time points after treatment.

*ZmbZIP92* expression profiles in various maize tissues and in response to ABA, NaCl, PEG-6000 (simulated drought stress), and 42 °C (heat stress) were determined via qRT-PCR. The generated data indicated that all tested abiotic stresses induced *ZmbZIP92* expression, with a rapid initial increase followed by a gradual decrease (Figure 2B–E). In terms of tissue-specific expression, *ZmbZIP92* transcript abundance was relatively high in roots, leaves, embryos, and endosperm, which was in contrast to the low transcript abundance in the stem at the eight-leaf stage and in silk at the flowering stage (Figure 2A).

### Nuclear localization and transcriptional self-activation analysis of *ZmbZIP92*

**Figure 3.**
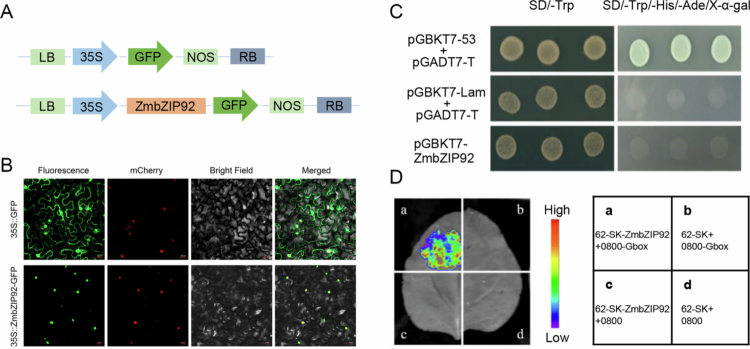
Identification and functional analysis of the transcription factor ZmbZIP92. (A) Schematic diagram of the construction of recombinant fluorescence vector 35S::GFP. (B) Subcellular localization of 35S::ZmbZIP92-GFP fusion protein in transiently transformed tobacco leaves. (Images acquired by laser scanning confocal microscopy). (C) Transcriptional activation assays. Yeast cells containing different plasmids were screened for growth on SD/-Trp and SD/-Trp/-His/-Ade/X-*α*-gal media. (D) The dual-LUC assay for *ZmbZIP92* with G-box cis-acting elements. G-box-0800, mG-box-0800, pGreenII 62-SK empty vector, and *ZmbZIP92*-62-SK were transiently expressed in *N. benthamiana*. The LUC signal was captured at 72 h post-transfection. The LUC/REN ratio indicating the relative luciferase activity in the dual-LUC assay.

To determine the subcellular localization and molecular properties of *ZmbZIP92*, we fused its coding sequence without the stop codon to the GFP-encoding sequence in an expression vector (Figure 3A) for the subsequent transient expression in *N. benthamiana* leaves. Green fluorescence due to the ZmbZIP92-GFP fusion protein was localized to the nucleus of tobacco leaf cells and its fluorescence signal highly overlapped with the spatial distribution of the Nuclear Localization Signal-mCherry-labeled nuclear region (Figure 3B), whereas the fluorescence of GFP alone was detected in the nucleus and cell membrane. Hence, *ZmbZIP92* is a nuclear protein.

To analyze the transcriptional self-activation of *ZmbZIP92*, its full-length coding sequence was cloned into the pGBKT7 vector, which was then inserted into yeast cells. Colonies that grew on solid SD/−Trp medium were spotted onto solid SD/−Trp/−His/−Ade selection medium containing X-*α*-gal in plates. Yeast cells carrying pGBKT7-ZmbZIP92, like the negative control, failed to grow on the selection medium (Figure 3C), reflecting a lack of transcriptional self-activation under experimental conditions.

### *ZmbZIP92* bound specifically to the G-box element

The G-box (core sequence: CACGTG) is a conserved cis-regulatory element that serves as a binding site for bZIP transcription factors.^29^ To determine whether *ZmbZIP92* binds specifically to this cis-regulatory element, recombinant vectors pGreenII 62-SK-ZmbZIP92 and pGreenII 0800-G-box were constructed for a dual-luciferase reporter assay. Significant luminescence was detected for the experimental group (62-SK-ZmbZIP92 and 0800-G-box) (Figure 3D), but not for any of the control groups (62-SK and 0800-G-box, 62-SK*-ZmbZIP92* and 0800, and 62-SK and 0800), indicating that *ZmbZIP92* binds specifically to the G-box element.

### *ZmbZIP92* overexpression enhanced drought resistance in *Arabidopsis*

Transgenic *Arabidopsis* plants overexpressing *ZmbZIP92* under the control of the CaMV 35S promoter were generated. Three independent transgenic lines were identified by PCR and qRT-PCR, with the highest *ZmbZIP92* expression level detected in line L5 (Figure S1A, B). Lines L4, L5, and L10 were selected for subsequent analyze on the basis of GUS staining results confirming that they were transgenic plants (Figure S1C).

**Figure 4.**
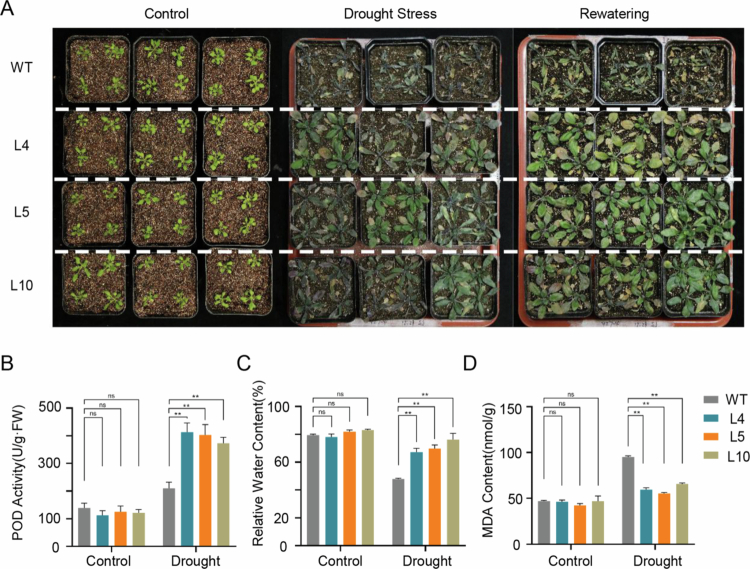
Enhanced drought tolerance in *ZmbZIP92*-overexpressing *Arabidopsis* lines. (A) Representative phenotypes of wild-type (WT) and three independent *ZmbZIP92*-overexpressing lines (L4, L5, L10) after 14 days of drought stress followed by 3 days of re-watering. (B-D) Physiological indices of WT and transgenic lines under drought conditions: Peroxidase (POD) activity, relative water content (RWC), and malondialdehyde (MDA) content in leaves. Data are presented as means ± SD of three biological replicates. Statistical significance was determined by Student’s *t*-test (*, *P* < 0.05, **, *P* < 0.01).

Drought stress adversely affected both WT and *ZmbZIP92*-overexpressing plants (inhibited growth, wilting, and yellowing leaves) (Figure 4A). After rewatering, transgenic plants recovered nearly completely, which was in contrast to the highly impaired recovery of WT plants. These results reflect the enhanced drought tolerance of the transgenic plants.

**Figure 5.**
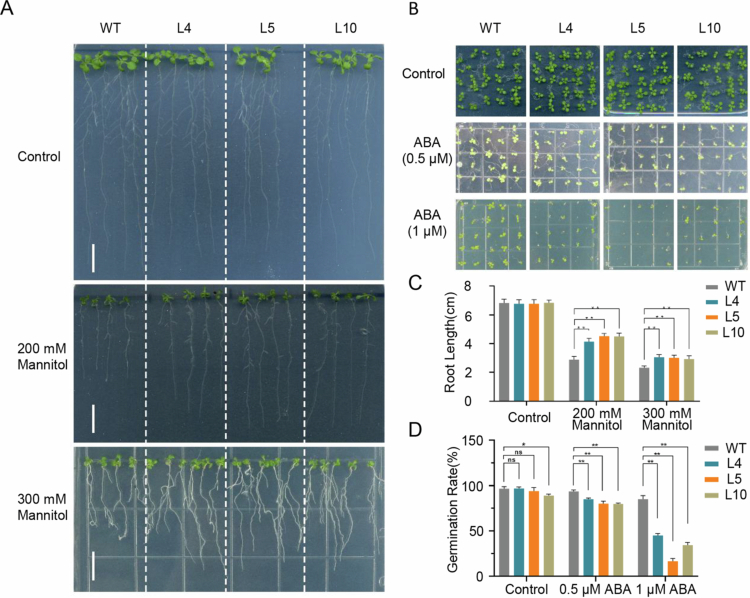
Phenotypic analysis of *ZmbZIP92*-overexpressing *Arabidopsis* under simulated drought and ABA treatment. (A) Root lengths of wild-type (WT) and transgenic lines grown on 1/2 MS medium supplemented with 0, 200, and 300 mM mannitol. (B) Germination of WT and transgenic lines on 1/2 MS medium containing 0, 0.5, or 1 μM ABA. (C) Statistical comparison of root lengths under the indicated mannitol concentrations. Roots were measured after 10 days of growth in the greenhouse. (D) Statistical analysis of *ZmbZIP92* overexpression lines and wild-type seed germination rates under ABA (0, 0.5, and 1 μM), respectively. *Arabidopsis* plants with two leaves after 7 days of growth were defined as successfully germinated. Each data point represents the mean of three independent biological replicates (mean ± SD, *, *P* < 0.05, **, *P* < 0.01).

Physiological analysis revealed that *ZmbZIP92* overexpression provided significant advantages under drought conditions. Compared with WT plants, transgenic plants had higher POD activities and RWC. Moreover, they accumulated less ROS and had lower MDA levels (Figure 4B–D; Figure S2). Under mannitol-simulated drought conditions, root growth was suppressed in both WT and transgenic plants (relative to that of untreated controls). However, in response to 200 and 300 mM mannitol treatments, *ZmbZIP92*-overexpressing plants developed significantly longer roots than WT plants, further supporting their improved tolerance to drought-like conditions (Figure 5A, C).

### Enhanced ABA sensitivity of *ZmbZIP92*-overexpressing *Arabidopsis*

Exogenous ABA applications rapidly induced *ZmbZIP92* expression. To assess whether this induction altered ABA responsiveness, we compared seed germination rates between WT and transgenic plants treated with 0.5 or 1 μM ABA. At both ABA concentrations, germination was inhibited significantly more for the *ZmbZIP92*-overexpressing lines (L4, L5, and L10) than for the WT control (Figure 5B, D). Thus, *ZmbZIP92* overexpression appeared to increase the sensitivity of *Arabidopsis* to ABA.

### *ZmbZIP92* mediated the drought response through the ABA signaling pathway

**Figure 6.**
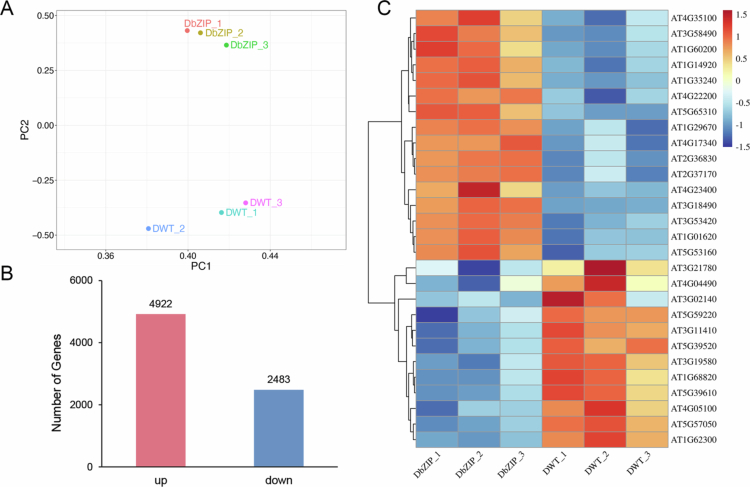
Comparative RNA-seq analysis of *Arabidopsis thaliana* wild-type and *ZmbZIP92*-overexpressing line. (A) Principal component analysis (PCA) of global gene expression profiles across all sequenced samples. The plot visualizes the overall variance and grouping pattern of samples based on their transcriptional profiles. (B) Number of differentially expressed genes (DEGs) between wild-type (WT) and *ZmbZIP92*-overexpressing (L5) *Arabidopsis* plants under drought stress (|log₂FC| > 1, FDR < 0.05). (C) Heatmap of expression patterns for 28 selected genes associated with ABA signaling and stress adaptation under drought conditions, illustrating coordinated up- or down-regulation in the transgenic line.

WT and L5 transgenic *Arabidopsis* plants exposed to drought stress underwent a transcriptome analysis. After confirming high sample consistency and sequencing data quality via PCA (Figure 6A), 7,405 DEGs were identified (Table S2). These DEGs consisted of 4,922 and 2,483 genes with upregulated and downregulated expression levels, respectively, in transgenic plants (Figure 6B). A set of 10 DEGs (five upregulated and five downregulated) was selected for further validation (Figure S3A, B). A total of 440 significantly enriched Gene Ontology (GO) terms from the molecular function, cellular component, and biological process categories were associated with the identified DEGs (Figure S4). These GO terms included key drought response-related terms, such as response to water deprivation (GO:0009414), ABA response (GO:0009737), defense response (GO:0006952), oxidative stress response (GO:0006979), salt stress response (GO:0009651), plant signal transduction (GO:0007165), and oxidation-reduction processes (GO:0055114). The subsequent Kyoto Encyclopedia of Genes and Genomes (KEGG) pathway analysis identified seven significantly enriched pathways, with plant hormone signal transduction revealed as the most enriched pathway (Figure S5).

By combining GO and KEGG analyses with protein functional annotations, 28 genes associated with ABA signaling and stress adaptations were identified. Cluster analysis indicated that 16 of these genes were upregulated and 12 were downregulated under drought conditions (Figure 6C). Functional annotations revealed that the upregulated genes included *ASPG1* (*AT3G18490*) and *RCAR3* (*AT5G53160*),^30^^,^^31^ which are implicated in ABA-related pathways, as well as *PIP2A* (*AT3G53420*), *PIP1;5* (*AT4G23400*), *PIP1C* (*AT1G01620*), *PIP2B* (*AT2G37170*), *PIP3* (*AT4G35100*), and *TIP2;2* (*AT4G17340*),^32–37^ which encode regulators of transmembrane water transport. By contrast, the downregulated genes (*AT3G02140*, *AT3G11410*, *AT3G19580*, *AT1G68820*, and *AT4G04490*) are reportedly associated with ABA-inhibitory responses.^38–42^ Decreases in the expression of these genes may contribute to ABA accumulation and enhanced drought tolerance (Table S3). Considered together, these findings suggest that *ZmbZIP92* may enhance drought tolerance by modulating ABA-related signaling pathways and coordinating the expression of key regulatory genes involved in stress adaptations and water homeostasis.

## Discussion

Maize, the most important grain, feed, and energy crop globally, is susceptible to drought stress, which can lead to severe yield and quality losses. There is increasing evidence of the involvement of multiple gene types in plant drought stress responses that alleviate the adverse effects of drought on growth and development.^43^^,^^44^ According to earlier research, bZIP transcription factors are key regulators of plant responses to abiotic stresses, including salinity, drought, and temperature extremes.^45^^,^^46^ In plants, bZIP transcription factors are encoded by a relatively large gene family. A total of 170 bZIP transcription factors (encoded by 125 genes) have been identified and grouped into 11 subfamilies according to their phylogenetic relationships with orthologs in *Arabidopsis* (75 bZIP family members) and rice (89 bZIP family members).^21^ Numerous studies have clarified bZIP transcription factor functions and the associated mechanisms underlying abiotic stress responses. For example, in tomato, *SlbZIP38* overexpression markedly decreases drought and salt tolerance, which may be attributed to decreased chlorophyll and free proline contents and increased MDA contents, suggesting that SlbZIP38 negatively regulates abiotic stress responses by modulating ABA signaling.^47^

In this study, the maize drought response‑related gene *ZmbZIP92* was identified according to its homology to OsbZIP62, which positively regulates drought tolerance in rice.^12^ The *Arabidopsis* homolog of *ZmbZIP92*, EEL, reportedly enhances drought tolerance.^48^ Our analysis of gene expression profiles indicated that *ZmbZIP92* is expressed in roots, leaves, and selected tissues, with transcript abundance peaking in roots and leaves at the three-leaf stage. As mentioned above, the expression of plant bZIP transcription factor genes can be induced by diverse abiotic stresses, with *ZmbZIP92* overexpression significantly improving phytohormone treatments.^10^^,^^45^ Earlier research demonstrated that the rice *bZIP* genes *OsbZIP62* and *OsbZIP16*, which are functional orthologs of *ZmbZIP92*, are transcriptionally activated by several abiotic stresses, such as drought and high salinity, as well as exogenous ABA.^12^^,^^49^ We also examined the effects of multiple stress treatments (200 mM NaCl, 20% PEG-6000, 42 °C, and 100 μM ABA) on *ZmbZIP92* expression. Our findings showed that *ZmbZIP92* expression was significantly induced by various abiotic stresses and exogenously applied ABA, but especially by drought and ABA. Hence, this gene may encode a protein with crucial functions during abiotic stress responses. The nuclear localization of *ZmbZIP92* further supports its identification as a canonical bZIP transcription factor. Transcriptional self-activation is a common feature of transcription factors. However, transcriptional self-activation of ZmbZIP92 was undetectable in yeast. Interestingly, similar findings were reported for OsbZIP62 and OsbZIP46.^12^^,^^50^ Although full-length OsbZIP62 cannot activate transcription, its N-terminal fragment can if the C-terminal domain is absent.^12^ Similarly, full-length OsbZIP46 does not activate transcription, but removing part of its C-terminal leads to detectable transcriptional activation.^50^ Notably, most bZIP transcription factors are transcriptional activators, including BhbZIP58, CsbZIP55, and CsbZIP65.^51^^,^^52^ Specifically, *ZmbZIP92* has characteristics analogous to those of OsbZIP62 and OsbZIP46, implying that they may have a common conserved regulatory mechanism.

*ZmbZIP92*-overexpressing transgenic *Arabidopsis* plants were obtained to clarify the *ZmbZIP92* function under drought conditions. Mannitol treatments promoted root elongation in transgenic plants, with the subsequent water deficit stress assays revealing a significant increase in drought tolerance. Thus, *ZmbZIP92* expression appears to be important for plant adaptive responses to drought stress. The enhanced stress tolerance of transgenic plants was reflected by increased POD activity and RWC along with a decrease in MDA contents. Collectively, these findings indicate that *ZmbZIP92* overexpression confers improved drought tolerance because of the associated positive regulation of drought stress responses. Therefore, *ZmbZIP92* functions as a transcription factor that positively regulates drought stress responses; the biological functions of ZmbZIP92 and its rice ortholog OsbZIP62 are conserved.^12^ Transcription factors mainly modulate the expression of downstream target genes by binding to their promoter regions, thereby enabling plants to adapt to various external stresses.^53^^,^^54^ We determined that ZmbZIP92 can bind to the G-box element of downstream target genes and mediate expression.^29^ Genes potentially regulated by *ZmbZIP92* were identified on the basis of RNA sequencing data. Moreover, according to significantly enriched GO terms, the 7,405 identified DEGs were associated with responses to ABA, salinity stress, water deprivation, and oxidative stress. KEGG pathway analysis indicated that most DEGs were involved in the MAPK signaling pathway and plant hormone signal transduction. Hence, these DEGs may contribute to the adaptive response of *Arabidopsis* to drought stress. Among the identified upregulated DEGs, *AT3G18490* encodes an aspartic protease (ASPG1) that promotes drought tolerance in *Arabidopsis* through the ABA signaling pathway,^30^ while *AT5G53160* encodes an ABA receptor (PYL8) that positively regulates ABA signaling.^31^^,^^55^ The downregulated genes included *AT3G02140*, which encodes tandem MAC motif-containing protein 2; this protein contains two or more ABRE elements and is a key negative regulator of ABA and salt stress responses.^38^ Another downregulated gene, *AT3G11410*, encodes protein phosphatase 2CA, which negatively regulates ABA signaling.^42^^,^^56^ In addition, genes encoding proteins that regulate water transport, such as *PIP1D* (*AT4G23400*) and *PIP1C* (*AT1G01620*), had upregulated expression levels.^33^^,^^57^*ZmbZIP92* expression is significantly induced by the application of ABA, with the corresponding transgenic plants exhibiting increased sensitivity to exogenous ABA. This suggests that *ZmbZIP92* may not uniformly amplify all downstream effects of ABA signaling, thereby optimizing plant resource allocation under stress conditions.

Increased ABA sensitivity enables plants to initiate basic defense responses, such as stomatal closure, relatively early and rapidly.^18^^,^^58^ More importantly, after integrating transcriptomic data, we speculated that *ZmbZIP92* preferentially activates ABA-responsive genes directly related to stress adaptations, including genes related to water retention and ROS scavenging. Simultaneously, ZmbZIP92 may have limited effects on ABA signaling that strongly inhibits cell growth and expansion. This differential regulation of downstream ABA signaling pathways enables the plant to prioritize survival under drought conditions, thereby enhancing overall stress resistance without incurring excessive growth costs.^59^ However, the absence of direct binding evidence (e.g., ChIP‑qPCR and EMSA) makes it unclear whether the identified DEGs are directly targeted by *ZmbZIP92*. Alternatively, the observed changes in the expression of these genes may be due to indirect responses within the regulatory network. This possibility decreases the direct mechanistic evidence of ZmbZIP92 binding to the G‑box promoter element and activating downstream gene expression.

Although our analysis of heterologous expression in *Arabidopsis* suggests that *ZmbZIP92* participates in ABA‑dependent drought responses, whether this transcription factor functions similarly in maize remains to be determined. The distinct regulatory networks of monocot species may alter the molecular mechanism or target gene specificity of ZmbZIP92. Definitive functional validation is required in future loss‑ and gain‑of‑function analysis of maize.

## Conclusion

This study functionally characterized *ZmbZIP92* as a maize bZIP transcription factor. Phylogenetic analysis classified it as a Group I member in the bZIP family, while also revealing its homology to bZIP proteins from *Arabidopsis* and rice. Subcellular localization confirmed that *ZmbZIP92* is a nuclear protein. Moreover, *ZmbZIP92* expression was induced by abiotic stresses, including salinity and drought. Although the full-length protein lacked transcriptional self-activation, it was observed to bind specifically to the G-box cis-element. Under drought conditions, *Arabidopsis* plants overexpressing *ZmbZIP92* exhibited enhanced drought tolerance, characterized by significant increases in RWC and POD activity as well as decreased MDA accumulation. Furthermore, *ZmbZIP92*-overexpressing plants were more sensitive to ABA than WT plants. Molecular analysis revealed that in response to drought stress, *ZmbZIP92* overexpression modulated the expression of key ABA signaling pathway genes, concurrently upregulating positive regulators and downregulating negative regulators. In summary, the study data imply that *ZmbZIP92* positively regulates the maize drought stress response, while also providing important molecular insights relevant to deciphering the mechanisms mediating maize drought tolerance.

## Supplementary Material

Figure S1.tif Figure S1.tif

Figure S2.tif Figure S2.tif

Figure S3.tif Figure S3.tif

Figure S4.tif Figure S4.tif

Figure S5.tif Figure S5.tif

Table S1.xlsx Table S1.xlsx

Table S2.xlsx Table S2.xlsx

Table S3.xlsx Table S3.xlsx

## References

[1] Brodersen CR, Roddy AB, Wason JW, McElrone AJ. Functional status of xylem through time. Annu Rev Plant Biol. 2019;70:407–433. doi: 10.1146/annurev-arplant-050718-100455. 30822114

[2] Blum A. Osmotic adjustment is a prime drought stress adaptive engine in support of plant production. Plant Cell Environ. 2017;40(1):4–10. doi: 10.1111/pce.12800. 27417527

[3] Hussain HA, Hussain S, Khaliq A, Ashraf U, Anjum SA, Men SN, Wang LC. Chilling and drought stresses in crop plants: implications, cross talk, and potential management opportunities. Front Plant Sci. 2018;9:393. doi: 10.3389/fpls.2018.00393. 29692787 PMC5902779

[4] Meng S, Cao Y, Li HG, Bian Z, Wang DL, Lian CL, Yin WL, Xia XL. PeSHN1 regulates water-use efficiency and drought tolerance by modulating wax biosynthesis in poplar. Tree Physiol. 2019;39(8):1371–1386. doi: 10.1093/treephys/tpz033. 30938421

[5] Chen F, Chen L, Yan Z, Xu JY, Feng LL, He N, Guo ML, Zhao JX, Chen ZJ, Chen HQ, et al. Recent advances of CRISPR-based genome editing for enhancing staple crops. Front Plant Sci. 2024;15:1478398. doi: 10.3389/fpls.2024.1478398. 39376239 PMC11456538

[6] Oyebamiji YO, Adigun BA, Shamsudin NA, Ikmal AM, Salisu MA, Malike FA, Lateef AA. Recent advancements in mitigating abiotic stresses in crops. Horticulturae. 2024;10(2):156. doi: 10.3390/horticulturae10020156.

[7] Sato H, Mizoi J, Shinozaki K, Yamaguchi-Shinozaki K. Complex plant responses to drought and heat stress under climate change. Plant J. 2024;117(6):1873–1892. doi: 10.1111/tpj.16612. 38168757

[8] Thilakarathne AS, Liu F, Zou ZW. Plant signaling hormones and transcription factors: key regulators of plant responses to growth, development, and stress. Plants-Basel. 2025;14(7):1070. doi: 10.3390/plants14071070. 40219138 PMC11990802

[9] Yang YL, Xu Y, Feng BZ, Li PQ, Li CQ, Zhu CY, Ren SN, Wang HL. Regulatory networks of bZIPs in drought, salt and cold stress response and signaling. Plant Sci. 2025;352:112399. doi: 10.1016/j.plantsci.2025.112399. 39874989

[10] Liu HT, Tang X, Zhang N, Li SG, Si HJ. Role of bZIP transcription factors in plant salt stress. Int J Mol Sci. 2023;24(9):7893. doi: 10.3390/ijms24097893. 37175598 PMC10177800

[11] Yoon S, Lee DK, Yu IJ, Kim YS, Choi YD, Kim JK. Overexpression of the *OsbZIP66* transcription factor enhances drought tolerance of rice plants. Plant Biotechnol Rep. 2017;11(1):53–62. doi: 10.1007/s11816-017-0430-2.

[12] Yang SQ, Xu K, Chen SJ, Li TF, Xia H, Chen L, Liu HY, Luo LJ. A stress-responsive bZIP transcription factor OsbZIP62 improves drought and oxidative tolerance in rice. BMC Plant Biol. 2019;19(1):260. doi: 10.1186/s12870-019-1872-1. 31208338 PMC6580479

[13] Lee YH, Song SI. OsbZIP62 positively regulates drought and salt stress tolerance and ABA signaling in rice. J Plant Biol. 2023;66(2):123–133. doi: 10.1007/s12374-022-09373-2.

[14] Liu CT, Mao BG, Ou SJ, Wang W, Liu LC, Wu YB, Chu CC, Wang XP. OsbZIP71, a bZIP transcription factor, confers salinity and drought tolerance in rice. Plant MolBiol. 2014;84(1-2):19–36. doi: 10.1007/s11103-013-0115-3. 23918260

[15] Yang JH, Qu X, Li T, Gao YX, Du HA, Zheng LJ, Ji MC, Zhang PF, Zhang Y, Hu JX, et al. HY5-HDA9 orchestrates the transcription of *HsfA2* to modulate salt stress response in *Arabidopsis*. J Integr Plant Biol. 2023;65(1):45–63. doi: 10.1111/jipb.13372. 36165397

[16] Matiolli CC, Tomaz JP, Duarte GT, Prado FM, Del Bem LEV, Silveira AB, Gauer L, Corrêa LGG, Drumond RD, Viana AJC, et al. The Arabidopsis bZIP gene AtbZIP63 is a sensitive integrator of transient abscisic acid and glucose signals. Plant Physiol. 2011;157(2):692–705. doi: 10.1104/pp.111.181743. 21844310 PMC3192551

[17] Kaya C, Ugurlar F, Adamakis IDS. Epigenetic modifications of hormonal signaling pathways in plant drought response and tolerance for sustainable food security. Int J Mol Sci. 2024;25(15):23. doi: 10.3390/ijms25158229 PMC11311266 39125799

[18] Verma V, Ravindran P, Kumar PP. Plant hormone-mediated regulation of stress responses. BMC Plant Biol. 2016;16 86. doi: 10.1186/s12870-016-0771-y. 27079791 PMC4831116

[19] Aslam MM, Waseem M, Jakada BH, Okal EJ, Lei ZL, Saqib HSA, Yuan W, Xu WF, Zhang Q. Mechanisms of abscisic acid-mediated drought stress responses in plants. Int J Mol Sci. 2022;23(3):1084. doi: 10.3390/ijms23031084. 35163008 PMC8835272

[20] Liu H, Song SB, Zhang H, Li YH, Niu LJ, Zhang JH, Wang W. Signaling transduction of ABA, ROS, and Ca2+ in plant stomatal closure in response to drought. Int J Mol Sci. 2022;23(23):14824. doi: 10.3390/ijms232314824. 36499153 PMC9736234

[21] Wei KF, Chen J, Wang YM, Chen YH, Chen SX, Lin YN, Pan S, Zhong XJ, Xie DX. Genome-wide analysis of bZIP-encoding genes in maize. DNA Res. 2012;19(6):463–476. doi: 10.1093/dnares/dss026. 23103471 PMC3514857

[22] Ma HZ, Liu C, Li ZX, Ran QJ, Xie GN, Wang BM, Fang S, Chu JF, Zhang JR. ZmbZIP4 contributes to stress resistance in maize by regulating ABA synthesis and root development. Plant Physiol. 2018;178(2):753–770. doi: 10.1104/pp.18.00436. 30126870 PMC6181033

[23] He L, Wu ZX, Wang XHY, Zhao CJ, Cheng DJ, Du CH, Wang HY, Gao Y, Zhang RJ, Han JA, et al. A novel maize F-bZIP member, ZmbZIP76, functions as a positive regulator in ABA-mediated abiotic stress tolerance by binding to ACGT-containing elements. Plant Sci. 2024;341:111952. doi: 10.1016/j.plantsci.2023.111952. 38072329

[24] Ying S, Zhang DF, Fu J, Shi YS, Song YC, Wang TY, Li Y. Cloning and characterization of a maize bZIP transcription factor, ZmbZIP72, confers drought and salt tolerance in transgenic *Arabidopsis*. Planta. 2012;235(2):253–266. doi: 10.1007/s00425-011-1496-7. 21866346

[25] He RY, Zheng JJ, Chen Y, Pan ZY, Yang T, Zhou Y, Li XF, Nan XY, Li YZ, Cheng MJ, et al. QTL-seq and transcriptomic integrative analyses reveal two positively regulated genes that control the low-temperature germination ability of MTP-maize introgression lines. Theor Appl Genet. 2023;136(5):116. doi: 10.1007/s00122-023-04362-6. 37093290

[26] Koornneef M, Meinke D. The development of Arabidopsis as a model plant. Plant J. 2010;61(6):909–921. doi: 10.1111/j.1365-313X.2009.04086.x. 20409266

[27] Provart NJ, Alonso J, Assmann SM, Bergmann D, Brady SM, Brkljacic J, Browse J, Chapple C, Colot V, Cutler S, et al. 50 years of Arabidopsis research: highlights and future directions. New Phytol. 2016;209(3):921–944. doi: 10.1111/nph.13687. 26465351

[28] Kasuga M, Liu Q, Miura S, Yamaguchi-Shinozaki K, Shinozaki K. Improving plant drought, salt, and freezing tolerance by gene transfer of a single stress-inducible transcription factor. Nat Biotechnol. 1999;17(3):287–291. doi: 10.1038/7036. 10096298

[29] Jia HM, Xu YP, Deng YW, Xie YH, Gao ZS, Lang ZB, Niu QF. Key transcription factors regulate fruit ripening and metabolite accumulation in tomato. Plant Physiol. 2024;195(3):2256–2273. doi: 10.1093/plphys/kiae195. 38561990 PMC11213253

[30] Belda-Palazon B, Gonzalez-Garcia MP, Lozano-Juste J, Coego A, Antoni R, Julian J, Peirats-Llobet M, Rodriguez L, Berbel A, Dietrich D, et al. PYL8 mediates ABA perception in the root through non-cell-autonomous and ligand-stabilization-based mechanisms. Proc Natl Acad Sci U S A. 2018;115(50):E11857–E11863. doi: 10.1073/pnas.1815410115. 30482863 PMC6294950

[31] Yao X, Xiong W, Ye TT, Wu Y. Overexpression of the aspartic protease ASPG1 gene confers drought avoidance in Arabidopsis. J Exp Bot. 2012;63(7):2579–2593. doi: 10.1093/jxb/err433. 22268147 PMC3346222

[32] Verdoucq L, Grondin A, Maurel C. Structure-function analysis of plant aquaporin *At*PIP2;1 gating by divalent cations and protons. Biochem J. 2008;415(3):409–416. doi: 10.1042/BJ20080275. 18637793

[33] Weig A, Deswarte C, Chrispeels MJ. The major intrinsic protein family of Arabidopsis has 23 members that form three distinct groups with functional aquaporins in each group. Plant Physiol. 1997;114(4):1347–1357. doi: 10.1104/pp.114.4.1347. 9276952 PMC158427

[34] Kammerloher W, Fischer U, Piechottka GP, Schaffner AR. Water channels in the plant plasma membrane cloned by immunoselection from a mammalian expression system. Plant J. 1994;6(2):187–199. doi: 10.1046/j.1365-313X.1994.6020187.x. 7920711

[35] Chen Q, Liu RJ, Wu YR, Wei SW, Wang Q, Zheng YN, Xia R, Shang XL, Yu FF, Yang XY, et al. ERAD-related E2 and E3 enzymes modulate the drought response by regulating the stability of PIP2 aquaporins. Plant Cell. 2021;33(8):2883–2898. doi: 10.1093/plcell/koab141. 34015125 PMC8408458

[36] Hachez C, Laloux T, Reinhardt H, Cavez D, Degand H, Grefen C, De Rycke R, Inzé D, Blatt MR, Russinova E, et al. Arabidopsis SNAREs SYP61 and SYP121 coordinate the trafficking of plasma membrane Aquaporin PIP2;7 to modulate the cell membrane water permeability. Plant Cell. 2014;26(7):3132–3147. doi: 10.1105/tpc.114.127159. 25082856 PMC4145137

[37] Hachez C, Veljanovski V, Reinhardt H, Guillaumot D, Vanhee C, Chaumont F, Batoko H. The Arabidopsis abiotic stress-induced TSPO-related protein reduces cell-surface expression of the Aquaporin PIP2;7 through protein-protein interactions and autophagic degradation. Plant Cell. 2014;26(12):4974–4990. doi: 10.1105/tpc.114.134080. 25538184 PMC4311218

[38] Huang MD, Wu WL. Overexpression of *TMAC2*, a novel negative regulator of abscisic acid and salinity responses, has pleiotropic effects in Arabidopsis thaliana. Plant Mol Biol. 2007;63(4):557–569. doi: 10.1007/s11103-006-9109-8. 17195036

[39] Drechsel G, Raab S, Hoth S. Arabidopsis zinc-finger protein 2 is a negative regulator of ABA signaling during seed germination. J Plant Physiol. 2010;167(16):1418–1421. doi: 10.1016/j.jplph.2010.05.010. 20619483

[40] Pei LS, Peng L, Wan X, Xiong J, Liu ZB, Li XF, Yang Y, Wang JM. Expression pattern and function analysis of AtPPRT1, a novel negative regulator in ABA and drought stress responses in Arabidopsis. Int J Mol Sci. 2019;20(2):394. doi: 10.3390/ijms20020394. 30658512 PMC6358930

[41] Tanaka H, Osakabe Y, Katsura S, Mizuno S, Maruyama K, Kusakabe K, Mizoi J, Shinozaki K, Yamaguchi-Shinozaki K. Abiotic stress-inducible receptor-like kinases negatively control ABA signaling in Arabidopsis. Plant J. 2012;70(4):599–613. doi: 10.1111/j.1365-313X.2012.04901.x. 22225700

[42] Sheen J. Mutational analysis of protein phosphatase 2C involved in abscisic acid signal transduction in higher plants. Proc Natl Acad Sci U S A. 1998;95(3):975–980. doi: 10.1073/pnas.95.3.975. 9448270 PMC18643

[43] Vadez V, Grondin A, Chenu K, Henry A, Laplaze L, Millet EJ, Carminati A. Crop traits and production under drought. Nat Rev Earth Environ. 2024;5:211–225. doi: 10.1038/s43017-023-00514-w.

[44] Yang ZR, Qin F. The battle of crops against drought: genetic dissection and improvement. J Integr Plant Biol. 2023;65(2):496–525. doi: 10.1111/jipb.13451. 36639908

[45] Guo ZL, Dzinyela R, Yang LM, Hwarari D. bZIP transcription factors: structure, modification, abiotic stress responses and application in plant improvement. Plants-Basel. 2024;13(15):2058. doi: 10.3390/plants13152058. 39124175 PMC11313983

[46] Joo H, Baek W, Lim CW, Lee SC. Post-translational modifications of bZIP transcription factors in abscisic acid signaling and drought responses. Curr Genomics. 2021;22(1):4–15. doi: 10.2174/1389202921999201130112116. 34045920 PMC8142349

[47] Pan YL, Hu X, Li CY, Xu X, Su CG, Li JH, Song HY, Zhang XG, Pan Y. *SlbZIP38*, a tomato bZIP family gene downregulated by abscisic acid, is a negative regulator of drought and salt stress tolerance. Genes. 2017;8(12):402. doi: 10.3390/genes8120402. 29261143 PMC5748720

[48] Baek D, Kim WY, Cha JY, Park HJ, Shin G, Park J, Lim CJ, Chun HJ, Li N, Kim DH, et al. The GIGANTEA-ENHANCED EM LEVEL complex enhances drought tolerance via regulation of abscisic acid synthesis. Plant Physiol. 2020;184(1):443–458. doi: 10.1104/pp.20.00779. 32690755 PMC7479899

[49] Chen H, Chen W, Zhou JL, He H, Chen LB, Chen HD, Deng XW. Basic leucine zipper transcription factor OsbZIP16 positively regulates drought resistance in rice. Plant Sci. 2012;193-194:8–17. doi: 10.1016/j.plantsci.2012.05.003. 22794914

[50] Tang N, Zhang H, Li XH, Xiao JH, Xiong LZ. Constitutive activation of transcription factor OsbZIP46 improves drought tolerance in rice. Plant Physiol. 2012;158(4):1755–1768. doi: 10.1104/pp.111.190389. 22301130 PMC3320183

[51] Liu W, Wang M, Zhong M, Luo C, Shi SQ, Qian YL, Kang YY, Jiang B. Genome-wide identification of bZIP gene family and expression analysis of BhbZIP58 under heat stress in wax gourd. BMC Plant Biol. 2023;23(1):598. doi: 10.1186/s12870-023-04580-6. 38017380 PMC10685590

[52] Hua B, Liang F, Zhang WY, Qiao D, Wang PQ, Teng HF, Zhang ZP, Liu JX, Miao MM. The potential role of bZIP55/65 in nitrogen uptake and utilization in cucumber is revealed via bZIP gene family characterization. Plants (Basel). 2023;12(18):3228. doi: 10.3390/plants12183228. 37765392 PMC10537890

[53] Xiang Y, Sun XJ, Bian XL, Wei TH, Han T, Yan JW, Zhang AY. The transcription factor ZmNAC49 reduces stomatal density and improves drought tolerance in maize. J Exp Bot. 2021;72(4):1399–1410. doi: 10.1093/jxb/eraa507. 33130877

[54] Ma ZX, Zhu XR, Min YW, Zhao DX, Zhang XS, Yan HD, Fang X, Cai RH, Ma Q. Mutation of *ZmWRKY87* increases maize salt sensitivity by regulating *ZmPP2C4* expression. Plant Physiol Biochem. 2025;229(Pt A):110388. doi: 10.1016/j.plaphy.2025.110388. 40850304

[55] Saavedra X, Modrego A, Rodríguez D, González-García MP, Sanz L, Nicolás G, Lorenzo O. The nuclear interactor PYL8/RCAR3 of *Fagus sylvatica* FsPP2C1 is a positive regulator of abscisic acid signaling in seeds and stress. Plant Physiol. 2010;152(1):133–150. doi: 10.1104/pp.109.146381. 19889877 PMC2799352

[56] Yoshida T, Nishimura N, Kitahata N, Kuromori T, Ito T, Asami T, Shinozaki K, Hirayama T. ABA-*Hypersensitive germination3* encodes a protein phosphatase 2C (AtPP2CA) that strongly regulates abscisic acid signaling during germination among Arabidopsis protein phosphatase 2Cs. Plant Physiol. 2006;140(1):115–126. doi: 10.1104/pp.105.070128. 16339800 PMC1326036

[57] Mosa KA, Kumar K, Chhikara S, Musante C, White JC, Dhankher OP. Enhanced boron tolerance in plants mediated by bidirectional transport through plasma membrane intrinsic proteins. Sci Rep. 2016;6:21640. doi: 10.1038/srep21640. 26902738 PMC4763227

[58] Ullah A, Manghwar H, Shaban M, Khan AH, Akbar A, Ali U, Ali E, Fahad S. Phytohormones enhanced drought tolerance in plants: a coping strategy. Environ Sci Pollut Res. 2018;25(33):33103–33118. doi: 10.1007/s11356-018-3364-5. 30284160

[59] Khan N. Molecular insights into ABA-mediated regulation of stress tolerance and development in plants. Int J Mol Sci. 2025;26(16):29. doi: 10.3390/ijms26167872. PMC12387064 40869193

