# Supplementary figures and images for: Maize ZmbZIP92 transcription factor positively regulates drought tolerance in Arabidopsis
Source: Plant Signal Behav. 2026 Feb 26;21(1):2635681. doi: 10.1080/15592324.2026.2635681 (PMC12947587; doi:10.1080/15592324.2026.2635681)

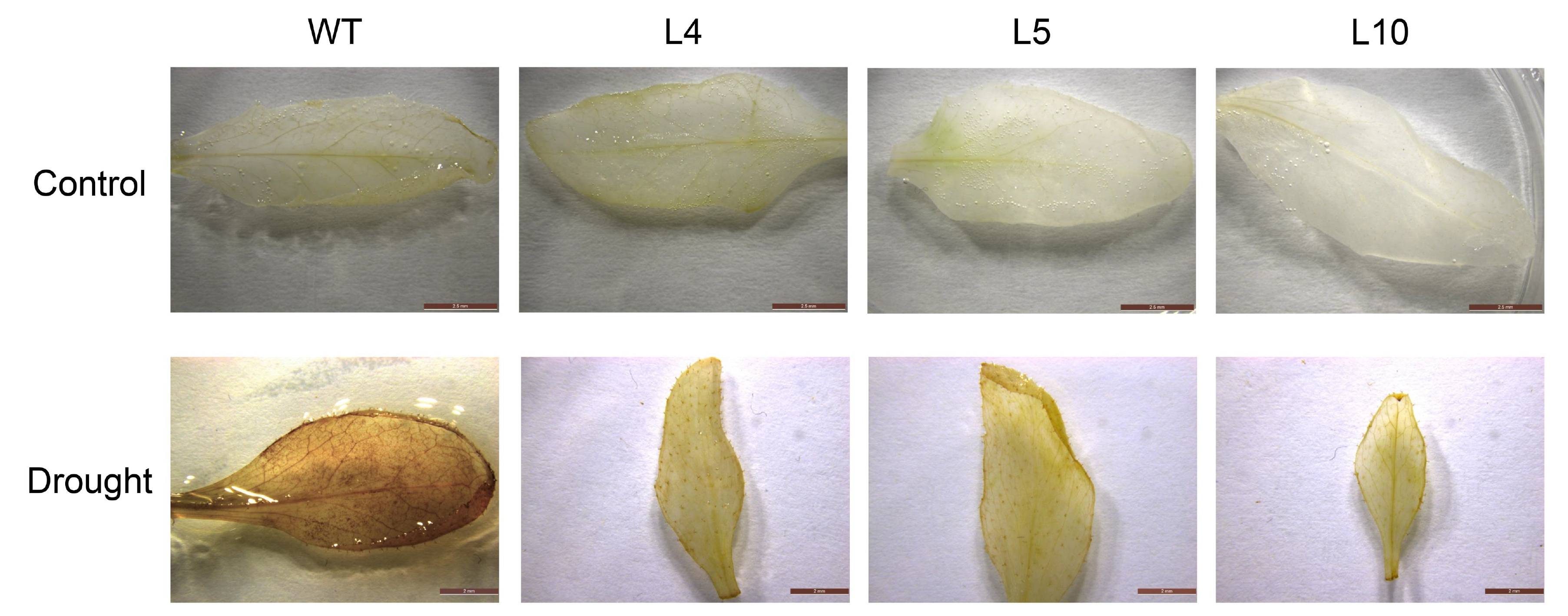

Supplement: Figure S1.tif [file KPSB_A_2635681_SM0055.tif]

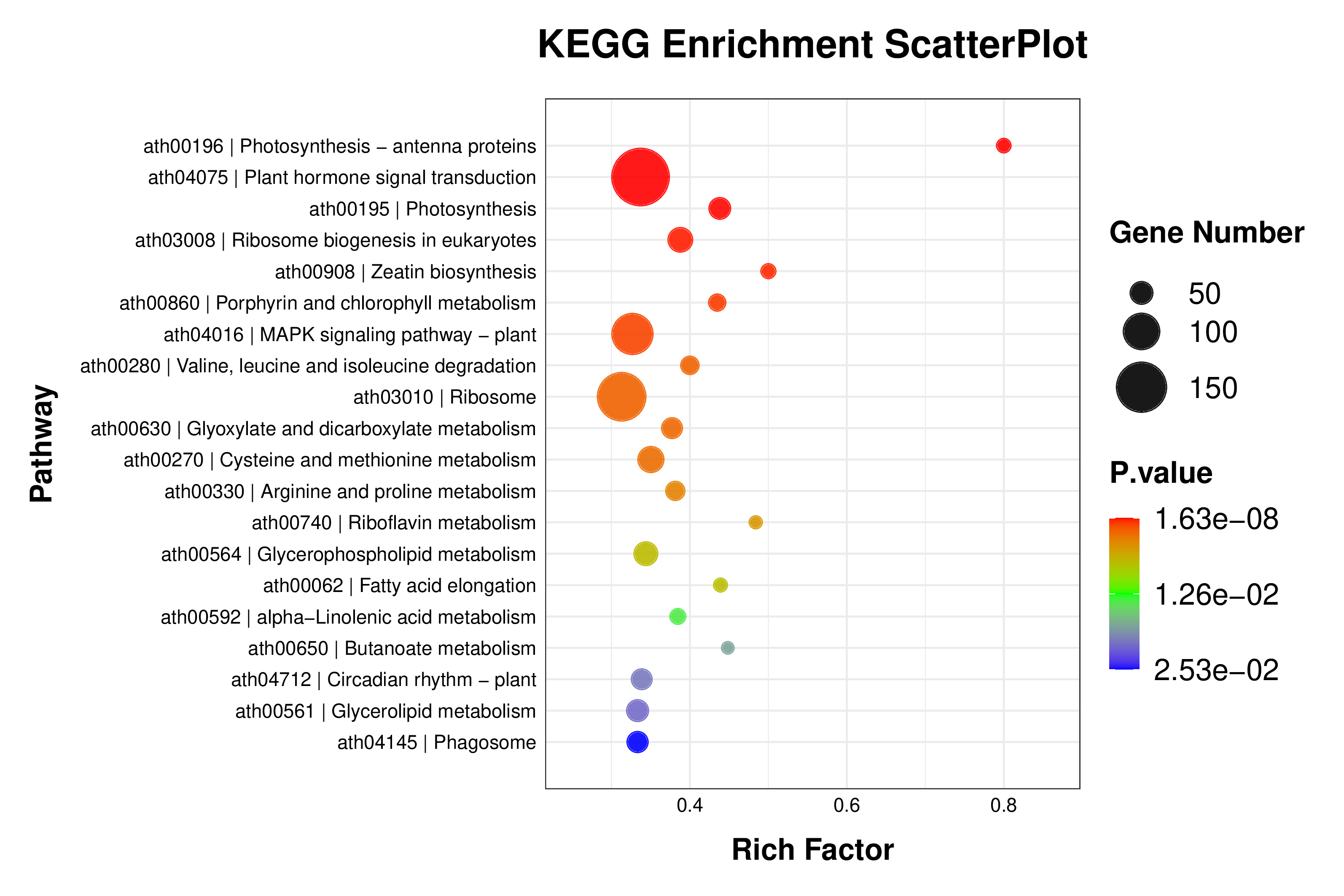

Supplement: Figure S2.tif [file KPSB_A_2635681_SM0048.tif]

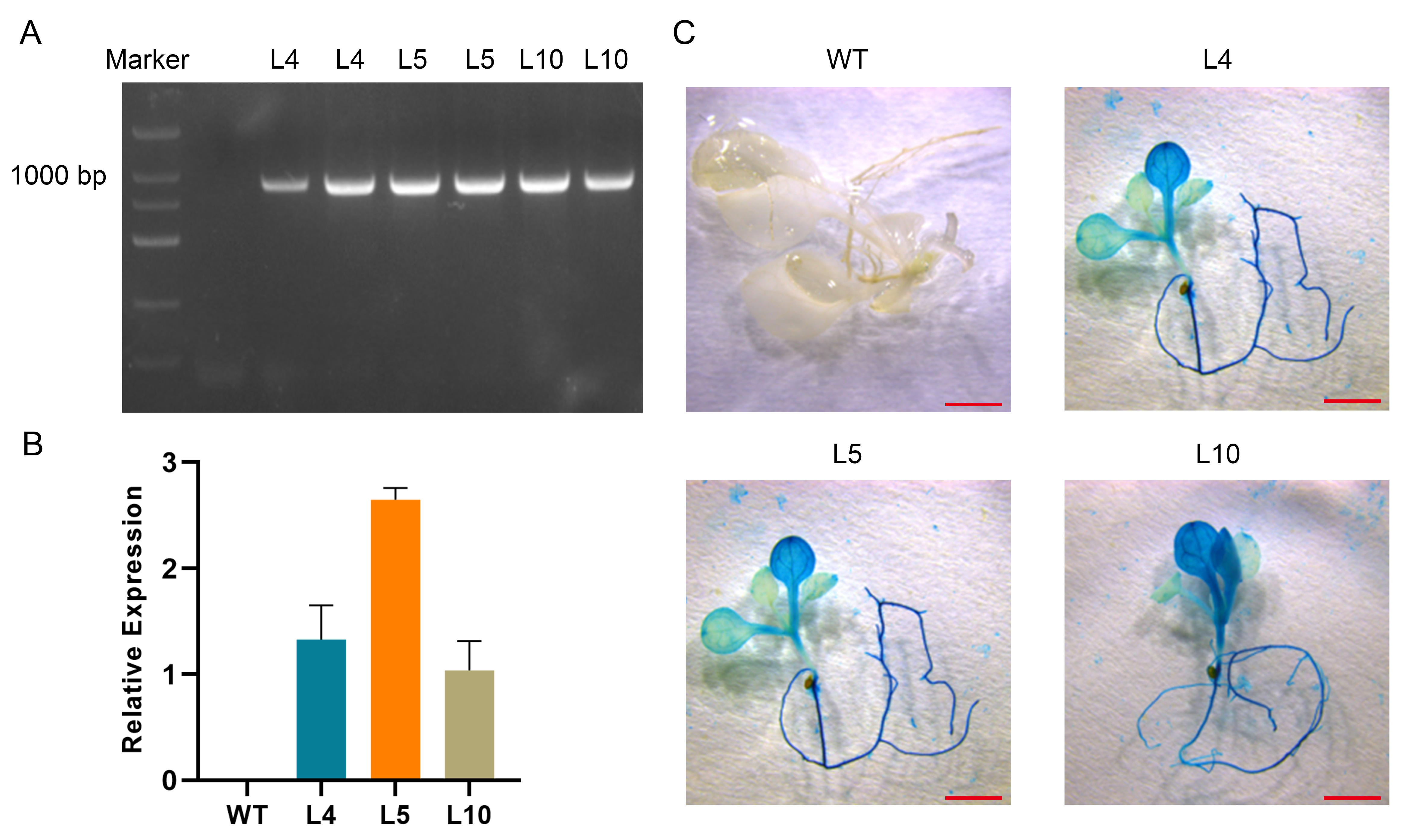

Supplement: Figure S3.tif [file KPSB_A_2635681_SM0049.tif]

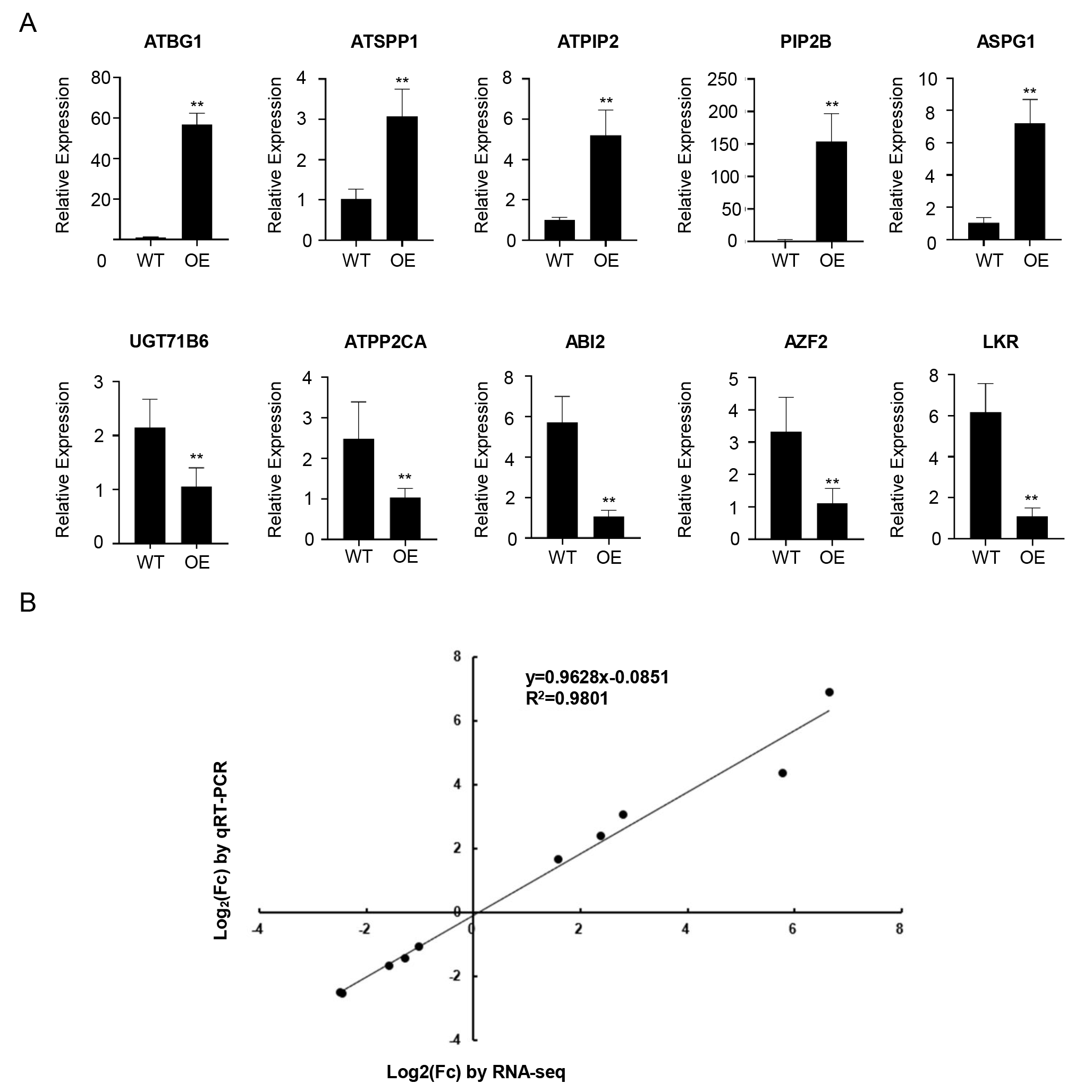

Supplement: Table S2.xlsx [file KPSB_A_2635681_SM0053.tif]
